# Pharmaceutical Induction of PGC-1*α* Promotes Retinal Pigment Epithelial Cell Metabolism and Protects against Oxidative Damage

**DOI:** 10.1155/2018/9248640

**Published:** 2018-11-05

**Authors:** Sangeeta Satish, Hannah Philipose, Mariana Aparecida Brunini Rosales, Magali Saint-Geniez

**Affiliations:** ^1^Graduate Medical Sciences, Boston University School of Medicine, Boston, MA 02118, USA; ^2^Schepens Eye Research Institute of Mass. Eye and Ear, Boston, MA 02114, USA; ^3^Department of Ophthalmology, Harvard Medical School, Boston, MA 02115, USA

## Abstract

Retinal pigment epithelium (RPE) dysfunction due to accumulation of reactive oxygen species and oxidative damage is a key event in the development of age-related macular degeneration (AMD). Here, we examine the therapeutic potential of ZLN005, a selective PGC-1*α* transcriptional regulator, in protecting RPE from cytotoxic oxidative damage. Gene expression analysis on ARPE-19 cells treated with ZLN005 shows robust upregulation of PGC-1*α* and its associated transcription factors, antioxidant enzymes, and mitochondrial genes. Energetic profiling shows that ZLN005 treatment enhances RPE mitochondrial function by increasing basal and maximal respiration rates, and spare respiratory capacity. In addition, ZLN005 robustly protects ARPE-19 cells from cell death caused by H_2_O_2_, ox-LDL, and NaIO_3_ without exhibiting any cytotoxicity under basal conditions. ZLN005 protection against H_2_O_2_-mediated cell death was lost in PGC-1*α*-silenced cells. Our data indicates that ZLN005 efficiently protects RPE cells from oxidative damage through selective induction of PGC-1*α* and its target antioxidant enzymes. ZLN005 may serve as a novel therapeutic agent for retinal diseases associated with RPE dystrophies.

## 1. Introduction

Age-related macular degeneration (AMD) is a multifactorial degenerative disease of the retina, retinal pigment epithelium (RPE), Bruch's membrane, and choroidal capillaries in the central, posterior region of the eye called the macula. Affecting 8.7% of the world, AMD is the third leading cause of vision loss in the geriatric population [1]. Risk factors of AMD [1] include age, smoking, and genetic variants.

These risk factors implicate increased oxidative stress as a key pathological process in the development of AMD [2]. The RPE, a polarized monolayer of pigmented cells, possesses important antioxidant enzymes and compounds to reduce oxidative stress and maintain retinal homeostasis [3]. Lying between the choroidal vasculature and the retina, the RPE plays a critical role in visual function by recycling the visual pigment, shuttling ions, metabolites, and macromolecules between the blood and retina and maintaining the composition of the subretinal space [4]. The RPE is exposed to high levels of reactive oxygen species (ROS), produced endogenously in the respiratory electron transport chain (ETC) [5] and through its function in phagocytosis and digestion of shed photoreceptor outer segments (POS) [6], and present exogenously in its location between the retina and choroid. To protect themselves from oxidative damage, the RPE employ antioxidant enzymes, such as superoxide dismutase (SOD1 and SOD2), catalase (CAT), and glutathione peroxidase (GPX), which are controlled by the nuclear respiratory factor 2 (NRF2 or NRE2L2) transcription factor [7].

However, with age, this balance between ROS generation and removal can be disrupted. Autofluorescent granules, called lipofuscin, are a byproduct of photoreceptor phagocytosis and begin occupying large volumes in the RPE with age. Irradiation of lipofuscin deposits break down its bisretinoid, lipid, and protein components causing photooxidative damage to the RPE [8]. The age-related loss of antioxidant enzymes decreases the RPE's defense against oxidative stress [9]. This loss plays a significant role in AMD pathogenesis as animal models with antioxidant enzyme knockouts have shown development of several AMD features [10, 11]. Smoking, a major AMD risk factor, induces further oxidative stress through the addition of ROS and free radicals [12]. This rising oxidative stress causes mitochondrial damage [13, 14] which leads to significant RPE dysfunction, inducing choroidal neovascularization or geographic atrophy observed in wet and dry AMD, respectively.

Peroxisome proliferator-activated receptor gamma coactivator 1-alpha (PGC-1*α*) and -beta (PGC-1*β*) are transcriptional coactivators that regulate mitochondrial function and metabolism in many tissues [15], including the retina [16–18]. To mediate their functions, the PGC-1 isoforms interact with transcription factors, such as estrogen-related receptor alpha (*ESRRA*) and nuclear respiratory factors 1 and 2 (NRF1 and NRF2) to control respiration, mitochondrial biogenesis, and expression of antioxidants [19].

So far, PGC-1*α* has been found to regulate expression of VEGF, control phagocytic and lysosomal function, enhance respiration and mitochondrial biogenesis, upregulate antioxidant genes in the RPE, and protect RPE from oxidative stress [16, 19, 20]. Due to its impact on oxidative stress, PGC-1*α* is being investigated as a therapeutic for numerous degenerative diseases, including AMD. A novel compound, ZLN005, was found to upregulate *PGC-1α* and its associated factors in the skeletal muscle [21]. Since ZLN005's effects in RPE have not been evaluated, this study focused on examining the impact of ZLN005 on RPE metabolism and antioxidant capacity.

## 2. Methods

### 2.1. Cell Culture

The human retinal pigment epithelia ARPE-19 cell line was obtained from the American Type Culture Collection (ATCC, Manassas, VA, USA). ARPE-19 cells were expanded in growth medium, consisting of Dulbecco's Modified Eagle Medium: Nutrient Mixture F-12 media (DMEM/F12, Thermo Fisher Scientific, Wilmington, DE, USA) supplemented with 10% fetal bovine serum (FBS, Atlanta Biologicals, Lawrenceville, GA), and 1% penicillin and streptomycin (PenStrep, Lonza, Walkersville, MD, USA), at 37°C and 10% CO_2_. Once cells reached confluency, they were maintained for five days in differentiation medium consisting of DMEM/F12, 1% FBS, and 1% PenStrep to allow for epithelial differentiation and polarization. A stock solution of 20 mM ZLN005 (Cayman Chemicals, Ann Arbor, MI, USA) was prepared in dimethyl sulfoxide (DMSO, D8418-Sigma, St. Louis, MO, USA) and diluted immediately before use in serum-free DMEM/F12. Cells were treated for up to 48 hours with 5–20 *μ*M ZLN005, and 0.05% DMSO in DMEM/F12 was used as the vehicle (veh) control.

### 2.2. RNA Collection and Quantitative PCR

Total RNA was collected using RNA-Bee (AMSbio, Lake Forest, CA, USA) and resuspended in distilled water. The concentration and purity of each sample was measured using the NanoDrop 2000 spectrophotometer (Thermo Fisher Scientific). Reverse transcription was performed with 1 *μ*g of RNA to produce the associated complimentary DNA (cDNA) using the IV Vilo Master Mix with ezDNase (Thermo Fisher Scientific). To measure the changes in gene expression, a quantitative polymerase chain reaction (qPCR) was performed using 3 *μ*l of cDNA template and the Power SYBR Green Master Mix (Thermo Fisher Scientific) on the LightCycler 480 (Roche Life Sciences, Indianapolis, IN, USA). Data was normalized to the mean expression of housekeeping genes (*CRYAB*, *PPIH*, *β-ACTIN*, and *HPRT1*) and quantified using the 2^−ΔΔCT^ method. The primer sequences used are listed in Table 1.

### 2.3. Protein Collection and Western Blot Analysis

Protein lysates were collected on ice using 1x sample lysis buffer (5 mM EDTA (pH 7), 2% SDS, 500 mM DTT, 10% sucrose, 100 mM Tris HCl (pH 6.8), 0.1% BPB) containing 2 mM phenylmethanesulfonyl fluoride (PMSF). Electrophoresis was carried out on 10% Mini-PROTEAN® TGX gels (Bio-Rad Laboratories, Hercules, CA, USA) with 40 *μ*l of protein and transferred to Immobilon-FL polyvinylidene difluoride membranes (EMD Millipore, Billerica, MA, USA). Dilutions of primary antibodies were made in 5% nonfat dry milk prepared in phospho-buffed saline, 0.1% (vol/vol) Tween-20 (PBS-T) or Tris-buffed saline, and 0.1% (vol/vol) Tween-20 (TBS-T). The membranes were incubated overnight at 4°C with mouse anti-human PGC-1*α* (1 : 250, EMD Millipore) and mouse anti-human *α*-tubulin (1 : 1000, Cell Signaling, Danvers, MA, USA). Following washes in PBS-T or TBS-T, the membranes were incubated with the secondary antibody (goat anti-mouse IgG20-HRP: 1 : 1000, Santa Cruz Biotechnology, Dallas, TX, USA) at room temperature for 1 hour. Membranes were exposed to the SuperSignal™ West Pico PLUS Chemiluminescent Substrate (Thermo Fisher Scientific) and developed on X-ray films (Kodak, Rochester, NY, USA). In each lane, the signal density of the PGC-1*α* band was measured using ImageJ [22] and normalized to the associated *α*-tubulin band signal density.

### 2.4. High-Resolution Respirometry

ARPE-19 were plated at 50,000 cells per well in V7-PS microplates (Seahorse Biosciences, Billerica, MA, USA) and cultured as described above. Following treatment, the media was replaced with the assay media: minimal DMEM containing 2 mM glutamine, 1 mM pyruvate, and 25 mM glucose (Seahorse Biosciences). Oxygen consumption rates (OCR) were measured using a XF-24 Extracellular Flux Analyzer (Seahorse Biosciences) under basal conditions and following the addition of the mitochondrial inhibitors from the XF Cell Mito Stress Kit (Seahorse Biosciences) at the following concentrations: 2.5 *μ*M oligomycin, 500 nM carbonyl cyanide-4-(trifluoromethoxy) phenylhydrazone (FCCP), and 2 *μ*M rotenone/antimycin A. All measured OCR were normalized to the number of cells quantified by DAPI staining at the end of the experiment, and the bioenergetics parameters were expressed as pmoles/min/cell.

### 2.5. MitoTracker Staining and Analysis

Cells were stained with 100 nM MitoTracker Orange CMTMRos (Thermo Fisher Scientific) in Hank's Balanced Salt Solution (HBSS, Thermo Fisher Scientific) for 30 minutes at 37°C, 10% CO_2_. Then, cells were washed with HBSS twice, fixed with 4% paraformaldehyde (VWR, Radnor, PA, USA), and permeabilized with 0.01% Triton X-100 (Sigma) for 5 min in PBS. Cell nuclei were counterstained with 1 *μ*g/ml of 4′,6-diamidino-2-phenylindole (DAPI, Sigma) before mounting. Fluorescent images were acquired on the Axioskop 2 mot plus microscope (Carl Zeiss Microscopy, Thornwood, NY, USA) with the AxioVision 4.8 software (Carl Zeiss Microscopy) at a set exposure of 600 ms. Median fluorescence intensity (MFI) for each image was quantified using the Adobe Photoshop CS6 software and normalized to total area of image in mm^2^.

### 2.6. Quantification of Mitochondria Superoxide Production

Cells were washed with HBSS and detached using Trypsin-EDTA. They were then resuspended at a concentration of 300,000 cells in 1.8 ml of 5 *μ*M MitoSox Red mitochondrial superoxide indicator (Thermo Fisher) in HBSS and incubated at 37°C, 5% CO_2_ for 30 minutes. The suspended cells are plated in a 96-well plate, and the associated fluorescence was measured as a function of superoxide levels produced by the mitochondria.

### 2.7. Cell-Death Assay

Cells were pretreated with 10 *μ*M ZLN005 prepared in serum-free, phenol-free DMEM for 24 hours before treatment with 500–1000 *μ*M hydrogen peroxide (H_2_O_2_, Sigma, 18 hours), 2–3 mg/ml sodium iodate (NaIO_3_, Sigma, 24 hours), or 100 *μ*g/ml oxidized low-density lipoprotein (ox-LDL, Alfa-Aesar, Haverhill, MA, USA, 48 hours). The supernatant media was collected and centrifuged to remove cells and debris. Cell death was quantified by the release of lactate dehydrogenase (LDH) from the cytoplasm of damaged cells into the media using the CytoTox 96® Non-Radioactive Cytotoxicity Assay (Promega Corp., Madison, WI, USA), which quantifies LDH levels as an optical density (OD) at 490 nm. Background levels of LDH present in the medium alone were measured, averaged, and subtracted from all other samples. Basal levels of LDH (0% cell death), measured in the supernatant of vehicle only treated cells, and maximal levels of LDH (100% cell death, total kill), measured by inducing complete cell death with 1x lysis buffer (Promega Corp.), were averaged and used to calculate percentage cell death using the following equation:

(1) $$\begin{array}{c} \%cell\;death=\frac{{{OD}_{490}}_{sample}-{{OD}_{490}}_{basal\;LDH}}{{{OD}_{490}}_{maximal\;LDH}}\times 100. \end{array}$$

### 2.8. Statistical Analysis

Statistical analysis was carried out by GraphPad Prism 5 software (GraphPad Software Inc., La Jolla, CA, USA). Data is presented as mean ± SEM. Student's *t*-test was used to calculate statistical significance between 2 groups. For more than 2 groups, one-way ANOVA followed by Dunnet's multiple comparison test was used. Statistical significance is denoted by the following: ns *P* > 0.05, ^∗^*P* < 0.05, ^∗∗^*P* < 0.01, ^∗∗∗^*P* < 0.001, and ^∗∗∗∗^*P* < 0.0001.

## 3. Results

### 3.1. ZLN005 Upregulates PGC-1*α* in Human RPE and Enhances RPE-Specific Genes and Mitochondrial Genes

**Figure 1:**
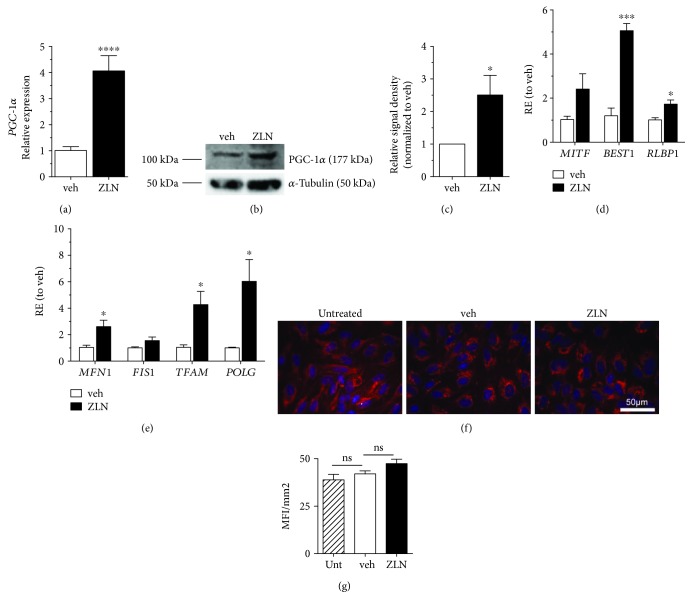
ZLN005 upregulates PGC-1*α* and its downstream mitochondrial gene targets in ARPE-19 cells. (a) Relative gene expression (RE) of *PGC-1α* in ARPE-19 treated for 48 hours with 10 *μ*M ZLN005 (ZLN, *n* = 5) compared to vehicle (veh, *n* = 4). (b) Representative protein blot of PGC-1*α* and *α*-tubulin bands and (c) quantification showed upregulation of PGC-1*α* protein expression with ZLN005 treatment (all conditions, *n* = 4). (d) RPE-specific genes, *BEST1* and *RLBP1*, are upregulated with ZLN005 treatment (veh, *n* = 4 for all genes; ZLN, *n* = 5 for all genes). (e) Treatment with 10 *μ*M ZLN005 increases the mitochondrial dynamic gene, *MFN1*, and replication genes, *TFAM* and *POLG* (veh, *n* = 4 for all genes; ZLN, *n* = 5 for all genes). (f, g) These changes do not impact mitochondrial morphology, as observed through (f) MitoTracker imaging (red) and (g) quantification in median fluorescence intensity (MFI) per mm^2^ (Unt, *n* = 3; veh, *n* = 2; ZLN, *n* = 3). All gene expression data were analyzed using Student's *t*-test. Statistical significance is represented as follows: ^ns^*P* > 0.05, ^∗^*P* < 0.05, ^∗∗^*P* < 0.01, ^∗∗∗^*P* < 0.001, and ^∗∗∗∗^*P* < 0.0001.

ZLN005 upregulates *PGC-1α* in skeletal muscle myotubes, but not in hepatocytes [21]. Because ZLN005's action appears cell-specific, the effect of ZLN005 on ARPE-19 cells was investigated. Differentiated ARPE-19 cells were treated with 10 *μ*M of ZLN005 for 48 hours, and a significant increase in both PGC-1*α* mRNA (Figure 1(a)) and protein levels (Figures 1(b) and 1(c)) was observed. Maturing ARPE-19 induce expression of RPE-specific genes and allow for cell polarization which aid the in vitro culture in acquiring characteristic RPE phenotype and functions [19, 23]. Upon treatment with ZLN005, expression of the RPE-specific genes, *BEST1* and *RLBP1*, were increased (Figure 1(d)), indicating that ZLN005 treatment not only does not interfere with RPE morphology and function but also may improve RPE specification.

*PGC-1α* upregulation has been found to induce mitochondrial genes and to promote mitochondrial biogenesis in numerous cell types, including RPE [19]. As expected, ZLN005 increases expression of the mitochondrial dynamic gene, *MFN1*, and replication genes, *TFAM* and *POLG* (Figure 1(e)). To determine if this increase affected mitochondrial morphology, the cells were stained using MitoTracker CMTMRos and the images were quantified as median fluorescence intensity (MFI) per unit area (mm^2^). There was no significant difference noted in mitochondrial morphology (Figure 1(f)) and MFI/mm^2^ (Figure 1(g)), indicating that although mitochondrial replication genes are induced, mitochondrial mass was not significantly increased.

### 3.2. ZLN005 Enhances Oxidative Phosphorylation through Upregulation of OXPHOS Genes

**Figure 2:**
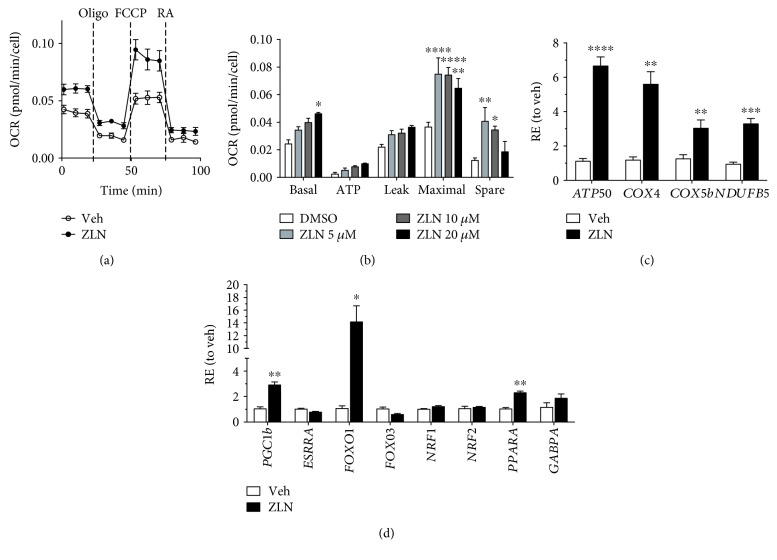
ZLN005 enhances mitochondrial respiration and upregulates OXPHOS targets. (a) Oxygen consumption rate (OCR) of ARPE-19 treated with 10 *μ*M ZLN005 measured by the Seahorse Bioanalyzer. Oligomycin (Oligo, 1 mM), FCCP (500 nM), and rotenone and antimycin A (RA, 2 mM) were injected at the marked intervals. (b) Bioenergetics profiling confirmed that 5–20 *μ*M ZLN005 efficiently increases basal and maximal RPE respiration. RPE spare capacity was significantly increased with 5 and 10 *μ*M ZLN005 (veh, *n* = 3; 5 *μ*M, *n* = 4, 10 *μ*M, *n* = 3, 20 *μ*M, *n* = 4, ANOVA). (c) OXPHOS genes, *ATP50*, *COX4*, *COX5b*, and *NDUFB5*, are significantly upregulated (veh, *n* = 4 for all genes; ZLN, *n* = 5 for all genes). (d) Relative expression of *PGC-1β*, *FOXO1*, and *PPARα* increase (veh, *n* = 4 for all genes; ZLN, *n* = 5 for all genes). All gene expression data was analyzed using Student's *t*-test. Statistical significance is represented as follows: ^∗^*P* < 0.05, ^∗∗^*P* < 0.01, ^∗∗∗^*P* < 0.001, and ^∗∗∗∗^*P* < 0.0001.

We have previously shown that *PGC-1α* overexpression in human RPE cells promotes mitochondrial function [19]. Therefore, we examined the effect of ZLN005 on ARPE-19 mitochondrial respiration and its associated gene targets. A representative OCR of ARPE-19 cells treated with 10 *μ*M ZLN005 shows enhanced oxidative respiration (Figure 2(a)). Quantification of the OCR for cells treated with 5–20 *μ*M ZLN005 for 24 hours showed an increase in the basal and maximal respiration rate. Most importantly, treatment with 5 and 10 *μ*M ZLN005 increases the spare respiration capacity, which allows cells to overcome unexpected and sudden changes in energy demand (Figure 2(b)). Based on this finding, we examined expression of respiratory gene targets, which have been found to be strongly induced with PGC-1*α* upregulation [19]. As expected, expression of OXPHOS genes, *ATP50*, *COX4*, *COX5b*, and *NDUFB5*, were robustly increased (Figure 2(c)). While expression of their commonly recognized upstream modulators, *ESRRA*, *NRF1*, and *GABPA*, were not found to be significantly increased (Figure 2(d)), metabolic modulators, *FOXO1* and *PPARα*, were upregulated with treatment. It is also interesting to note that ZLN005 increases expression of *PGC-1β* (Figure 2(d)), an isoform of *PGC-1α* that is usually suppressed in differentiated ARPE-19 [19].

### 3.3. ZLN005 Increases Expression of Antioxidant Enzymes and Reduces Prooxidant-Induced Cell Death in a PGC-1*α*-Dependent Manner

**Figure 3:**
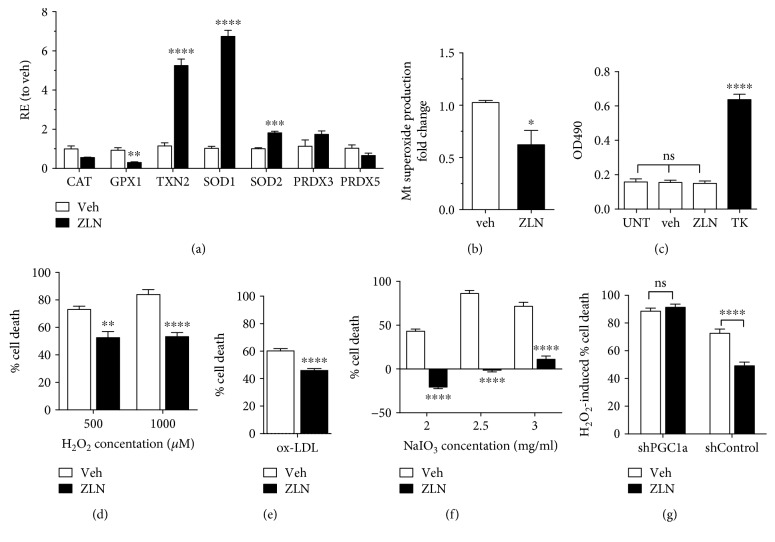
ZLN005 protects ARPE-19 cells from prooxidant-induced cell death through the upregulation of PGC-1*α*. (a) Treatment with 10 *μ*M ZLN005 increases mitochondrial antioxidant enzymes, *TXN2* and *SOD2.* Expression of the cytoplasmic enzyme, *SOD1,* is also enhanced, while *GPX1* is downregulated (veh, *n* = 4 for all genes; ZLN, *n* = 5 for all genes). (b) Accordingly, mitochondrial superoxide production is decreased after 24-hour treatment with ZLN005 (veh, *n* = 4 for all genes; ZLN, *n* = 5 for all genes). (c) 48 hours of exposure to 10 *μ*M ZLN005 (*n* = 6) does not increase LDH levels, measured as OD_490_, compared to untreated cells (UNT, *n* = 6) and vehicle only treatment (veh, *n* = 6). Total kill (TK, *n* = 6) of cells is achieved by treatment with 1x lysis buffer for 30 min. (d) Cell death induced by 18-hour exposure to 500 *μ*M and 1000 *μ*M H_2_O_2_ decreases significantly with pretreatment with 10 *μ*M ZLN005 (*n* = 6 for all conditions). (e) ZLN005 protects cells from cytotoxicity mediated by 100 *μ*g/ml ox-LDL (*n* = 6 for all conditions). (f) 24-hour pretreatment with 10 *μ*M ZLN005 protects against NaIO_3_-induced cell death in the differentiated ARPE-19. ZLN005 protection decreased LDH levels in prooxidant conditions below basal LDH levels when exposed to 2 and 2.5 mg/ml NaIO_3_ (veh, *n* = 5; ZLN, *n* = 6). (g) Cells lacking PGC-1*α* (shPGC-1*α*, *n* = 6) show a loss of the protective effect of 10 *μ*M ZLN005 upon exposure to 1000 *μ*M H_2_O_2_, while the effect is maintained in the associated control cells (shControl, *n* = 12).

Consistent with antioxidant upregulation by PGC-1*α* [19], ZLN005 treatment induced the key antioxidant enzymes, *SOD1*, *SOD2*, and *TXN2* (Figure 3(a)). A decrease in basal mitochondrial superoxide production was also observed (Figure 3(b)). Reduced superoxide production combined with increased *SOD2* is likely to reduce endogenous hydrogen peroxide levels which may explain the slight inhibition of *GPX1* expression observed [24] (Figure 3(a)). Together, this suggests that ZLN005 could efficiently protect RPE cells from pathological oxidative damage. To investigate this further, cells were exposed to three biologically relevant prooxidants: hydrogen peroxide (H_2_O_2_), oxidized low-density lipoprotein (ox-LDL), and sodium iodate (NaIO_3_). H_2_O_2_ is produced endogenously in RPE through respiration [5] and outer segment phagocytosis [6]. The pathological lipid, ox-LDL, increases with age and is found in AMD drusen [25, 26]. NaIO_3_, an oxidizing agent specifically toxic to RPE, is commonly used to replicate RPE dysfunction seen in geographic atrophy [27] by inducing mitochondrial oxidative damage [28].

First, we confirmed the lack of cytotoxic effect of treatment with 10 *μ*M ZLN005 by measuring LDH release under basal conditions (Figure 3(c)). Next, cells pretreated with 10 *μ*M ZLN005 for 24 hours were exposed to highly cytotoxic doses of AMD-associated prooxidant, and cell death was quantified. Our results demonstrate that, in all tested conditions, ZLN005 treatment significantly protected RPE cells from oxidant-induced cell death (Figures 3(d) and 3(f)), with an average decrease of 20% in H_2_O_2_ and ox-LDL-mediated cell death (Figures 3(d) and 3(e)), and a 70% decrease in NaIO_3_-mediated cell death (Figure 3(f)).

To determine if this protection against oxidative stress is dependent on ZLN005's upregulation of PGC-1*α*, ARPE-19 cells silenced for PGC-1*α* (shPGC-1*α*) and the associated control (shControl), previously generated by the lab (Rosales MAB et al. IOVS 2017; 58: ARVO Abstract 3014), were exposed to 1000 *μ*M H_2_O_2_. Pretreatment with ZLN005 was not able to protect shPGC-1*α* cells from H_2_O_2_-mediated cytotoxicity but caused a 24% decrease in shControl cell death (Figure 3(g)), indicating that PGC-1*α* is required for ZLN005 antioxidant function.

## 4. Discussion

Our results demonstrated that the small molecule ZLN005 stimulates the upregulation of the PGC-1*α* gene and protein in RPE. An associated increase in several downstream respiratory and mitochondrial gene targets were observed, leading to enhanced oxidative respiration. Furthermore, ZLN005 treatment increased the expression of antioxidant enzymes, decreased mitochondrial superoxide production, and conferred protection against prooxidant-induced cell death. This protective effect was found to be dependent on ZLN005 induction of PGC-1*α*, as protection was lost in cells lacking PGC-1*α* when exposed to H_2_O_2_.

While we show that ZLN005 cytoprotection depends on PGC-1*α*, the molecular mechanism of such induction is unclear. In other tissues, ZLN005 has been found to increase AMP-kinase activity [21] and SIRT1 expression [29], two known regulators of PGC-1*α* function. Furthermore, blockade of AMP-kinase activity was able to blunt ZLN005-dependent PGC-1*α* induction, suggesting AMP-kinase as the primary target of ZLN005, at least in L6 myotubes [21]. Whether ZLN005 transcriptional activity in RPE is also dependent on AMP-kinase remains to be determined, but nonetheless, we have established that PGC-1*α* is critical in inducing the downstream therapeutic effects of ZLN005.

ZLN005 also increased expression of PGC-1*α* gene targets that promote mitochondrial biogenesis and function. No changes in the mitochondrial structure and network were apparent at the time points studied. However, as mitochondrial turnover takes days, it is possible that following longer exposure, ZLN005 may increase mitochondrial mass. On the other hand, PGC-1*α* is known to promote mitophagy [30], and *FOXO1*, seen to be upregulated with ZLN005 treatment, has been found to target damaged mitochondria and induce mitophagy [31]. Therefore, it is also possible that ZLN005-dependent mitobiogenesis is offset by concomitant recycling, causing no change in mitochondrial mass, but enhancing mitochondrial function. ZLN005 treatment improved mitochondrial respiration measures by significantly increasing the basal and maximal respiratory rates. Most important, the spare respiratory capacity of RPE cells was increased with ZLN005 treatment. The spare respiratory capacity is a measure of the respiratory potential of a cell which allows cell survival during sudden changes in energy demands [32], such as energy deprivation induced by oxidative stress. An increased spare respiratory capacity has been associated with better protection against such fluctuations leading to decreased cell death [32, 33].

When examining potential upstream transcription factor expression responsible for these changes, *FOXO1* and *PPARα* were found to be upregulated with treatment. However, common transcription factors, such as *ESRRA* and *GABPA*, which were found to increase with PGC-1*α* overexpression [19], did not experience any change in expression with ZLN005 treatment. Indeed, these transcriptional factors were upregulated with exposure to high and potentially super-physiological levels of PGC-1*α*, whereas ZLN005 treatment induces only a 4-fold change of endogenous levels. The inability of this change to enhance expression of these common transcription factors could indicate a more complex interaction in place, and future work with ZLN005 could help elucidate these pathways more thoroughly.

Evaluating antioxidant activity, ZLN005 was found to upregulate several antioxidant enzymes and decrease mitochondrial superoxide production. These changes conferred protection against prooxidant-induced cell death. This study confirmed PGC-1*α* as the central mediator of ZLN005 antioxidant activity, as PGC-1*α* silencing led to a loss of ZLN005-induced protection against prooxidant-induced cell death. Upon exposure to NaIO_3_, ZLN005 pretreated cells had a dramatic reduction in LDH release. With 2 and 2.5 mg/ml, this reduction was associated with a negative percentage cell death, indicating that ZLN005 had diminished LDH release below the basal levels. NaIO_3_ has been found to induce mitochondrial superoxide levels [34] and mitochondrial network fragmentation [35], while decreasing mitochondrial membrane potential [36]. As shown in this study, ZLN005 decreases basal mitochondrial superoxide levels, upregulates OXPHOS targets that help maintain mitochondrial membrane potential and spare respiratory capacity, and increases mitochondrial gene levels. These mitochondrial specific effects of ZLN005 could lead to the enhanced protection observed against NaIO_3_. While similar processes are induced by H_2_O_2_ and ox-LDL, these oxidants also induce other nonmitochondrial cell-death mechanisms. For example, ox-LDL promotes NLPR3-caspase1 activation [26] and exogenous H_2_O_2_, while mimicking the effects of endogenous H_2_O_2_, will diffuse and drive multiple oxidant-dependent pathways in many cellular compartments [37]. This could explain the reduced protective effect of ZLN005 on H_2_O_2_- and ox-LDL-induced RPE cell death. The potential synergistic effects of ZLN005 with compounds that target different pathways could be investigated to elucidate mechanisms involved in RPE dysfunction and diminish prooxidant-induced cell death.

Due to its effects on antioxidant function and respiration, therapeutics that cause a gain of function in PGC-1*α* are being investigated for several diseases involving oxidative damage [21, 38]. Methods currently in use to overexpress PGC-1*α*, such as adenovirus, induce super-physiological expression of PGC-1*α* in tissue, which can sometimes lead to cell death [39]. Therefore, there is a need for a pharmaceutical that induces expression at physiologically relevant levels. The ability of ZLN005 to induce a physiological increase in PGC-1*α* protein levels (2 folds) and robustly enhance the cell antioxidant capacity makes this compound highly promising. While further study involving in vivo testing of ZLN005 in the eye is necessary, this current study has introduced ZLN005 as a potential therapeutic against AMD pathogenesis.

## 5. Conclusions

In summary, our study has shown that the small molecule ZLN005 increases PGC-1*α* expression in the human RPE. An induction of downstream respiratory, mitochondrial, antioxidant, and transcription factor targets was observed. ZLN005 treatment protected cells from cell death induced by three biologically relevant prooxidants. Most importantly, we have established PGC-1*α* as the critical mediator of ZLN005 antioxidant effects. Oxidative stress is a major cause of RPE dysfunction in AMD and there is a current need for therapeutics to combat this rise in oxidative damage. ZLN005's cytoprotective effects against AMD-relevant prooxidants suggest that this compound could be a potential therapeutic for ocular diseases, such as AMD.

## Figures and Tables

**Table 1:** Primer sequences used for gene expression experiments.

Gene symbol	Gene name	Forward sequence (5′–3′)	Reverse sequence (5′–3′)
*CRYAB*	Crystallin alpha B	GTTCTTCGGAGAGCACCTGTT	GAGAGTGCAGTGTCAAACCAG
*PPIH*	Peptidylprolyl isomerase H	CCCCAACAATAAGCCCAAG	CACCACCAAGAAGAAGGGAA
*HPRT1*	Hypoxanthine phosphoribosyltransferase 1	CCTGGCGTCGTGATTAGTGAT	AGACGTTCAGTCCTGTCCATAA
*β-Actin*	Beta-actin	CTGTCTGGCGGCACCACCAT	GCAACTAAGTCATAGTCCGC
PGC-1*α*	Peroxisome proliferator-activated receptor gamma coactivator 1 alpha	GTCACCACCCAAATCCTTAT	ATCTACTGCCTGGAGACCTT
PGC-1*β*	Peroxisome proliferator-activated receptor gamma coactivator 1 beta	CCACATCCTACCCAACATCAAG	CACAAGGCCGTTGACTTTTAGA
*ESRRA*	Estrogen-related receptor alpha	TATGGTGTGGCATCCTGTG	GTCTCCGCTTGGTGATCTC
*FOXO1*	Forkhead box O1	AAGAGCGTGCCCTACTTCAA	GCACACGAATGAACTTGCTG
*FOXO3*	Forkhead box O3	CTTCAAGGATAAGGGCGACA	AGTTCCCTCATTCTGGACCC
*NRF1*	Nuclear respiratory factor 1	GCTGATGAAGACTCGCCTTCT	TACATGAGGCCGTTTCCGTTT
*NRF2*	Nuclear factor, erythroid 2 like 2	TCCAGTCAGAAACCAGTGGAT	GAATGTCTGCGCCAAAAGCTG
*PPARα*	Peroxisome proliferator-activated receptor alpha	ATCGAATGTAGAATCTGCGGG	TCGCACTTGTCATACACCAG
*ATP5O*	ATP synthase subunit O, mitochondrial	TTTGAATCCCTATGTGAAGCGTT	CCTTGGGTATTGCTTAATCGACC
*COX4I1*	Cytochrome c oxidase subunit 4 isoform 1	GCACTGAAGGAGAAGGAGAAG	AACCGTCTTCCACTCGTTC
*COX5B*	Cytochrome c oxidase subunit 5B	GGAAGACCCTAATTTAGTCCCCT	CCAGCTTGTAATGGGCTCCAC
*NDUFB5*	NADH-ubiquinone oxidoreductase subunit B5	CACTCGCCTCGGATTTGG	CGCCTGTCATAGAATCTAGAAGG
*CAT*	Catalase	ACTTTGAGGTCACACATGACATT	CTGAACCCGATTCTCCAGCA
*GPX1*	Glutathione peroxidase 1	CCAGTCGGTGTATGCCTTCTC	GAGGGACGCCACATTCTCG
*TXN2*	Thioredoxin 2	TGATGACCACACAGACCTCG	ATCCTTGATGCCCACAAACT
*SOD1*	Superoxide dismutase 1	AGGGCATCATCAATTTCGAGC	GCCCACCGTGTTTTCTGGA
*SOD2*	Superoxide dismutase 2	CAGACCTGCCTTACGACTATGG	CGTTCAGGTTGTTCACGTAGG
*PRDX3*	Peroxiredoxin 3	GATTTCCCGAGACTACGGTG	GACGCTCAAATGCTTGATGA
*PRDX5*	Peroxiredoxin 5	AGTGAAGGAGAGTGGGCGTC	TTCAAACACCTCCACTGCTG
*MFN1*	Mitofusin 1	TGCCCTTCACATGGACAAAG	CTCTGTAGTGACATCTGTGCC
*FIS1*	Fission 1	TGACATCCGTAAAGGCATCG	CTTCTCGTATTCCTTGAGCCG
*TFAM*	Transcription factor A, mitochondrial	CCATATTTAAAGCTCAGAACCCAG	CTCCGCCCTATAAGCATCTTG
*POLG*	DNA polymerase gamma	GAAGGACATTCGTGAGAACTTCC	GTGGGGACACCTCTCCAAG
*MITF*	Microphthalmia-associated transcription factor	AGCCATGCAGTCCGAAT	ACTGCTGCTCTTCAGCG
*BEST1*	Bestrophin 1	GAATTTGCAGGTGTCCCTGT	ATCCTCCTCGTCCTCCTGAT
*RLBP1*	Retinaldehyde-binding protein 1	CACGCTGCCCAAGTATGATG	CCAGGACAGTTGAGGAGAGG

## Data Availability

The data used to support the findings of this study are available from the corresponding author upon request.
